# Inclusive leadership and financial–marketing decision-making in crises: gender diversity and brand resilience

**DOI:** 10.3389/fpsyg.2026.1730375

**Published:** 2026-02-18

**Authors:** Anam Javeed, Muhammad Yar Khan, Abdulrahman Alomair, Abdulaziz S. Al Naim

**Affiliations:** 1 Faculty of Society and Culture, Northumbria University, Newcastle upon Tyne, United Kingdom; 2 Department of Accounting and Finance, Effat University, Jeddah, Saudi Arabia; 3 Tenured Associate Professor, Department of Management Sciences, COMSATS University Islamabad, Islamabad, Pakistan; 4 Assistant Professor of Accounting, Department of Accounting, Business School, King Faisal University, Al-Ahsa, Saudi Arabia

**Keywords:** brand equity resilience, crisis leadership, gender diversity, institutional context, marketing investment

## Abstract

**Introduction:**

This paper explores how the gender and diversity of leadership, as well as institutional context, influence crisis strategies and brand equity resilience across the UK and Saudi Arabia.

**Methods:**

The study has used upper echelons and role congruity theories, a mixed-methods explanatory sequential design integrated survey data from 298 firms with interviews from 32 executives.

**Results:**

Structural equation modeling analysis indicated that female leadership and financial austerity were found to be positive drivers of marketing investment and empathetic communication which mediated the indirect relationship between these two factors and brand equity resilience. Gender-diverse leadership teams exhibited higher brand resilience than did single-gender teams and the institutional context moderated these relationships with the UK showing sharper gender differences rather than Saudi Arabia. Qualitative findings shed light on how gendered communication and context-specific norms influence decision-making in crises.

**Discussion:**

The results highlight that it is marketing and stakeholder communication to maintain activity, not austerity, that will protect brand equity. Organizations should promote a gender-balanced leadership, and empathetic communication to enhance the resilience of businesses in economic recessions.

## 1 Introduction

Economic recessions, such as the 2008 Global Financial Crisis and COVID-19, fragment financial stability and brand equity, calling for companies to maneuver through funding caps and continue building consumer confidence (Rego et al., 2022). Brand equity, which is the added value endowed by brand knowledge to customer response (Keller, 1993), acts as a strategic asset that enables corporations to survive downturns by retaining loyalty and providing long-term value. Strategic leadership is vital to protect this asset: Financial executives typically favor capital preservation, while marketing executives favor brand investment (Cohen et al., 2008). Cross-functional leadership in the context of crisis decision making This tension between is-and-ought situations indicating a need for cross-functional leadership in crisis decision-making.

The upper echelons theory (Hambrick and Mason, 1984) upper echelons theory argues that executives’ demographic characteristics and psychological traits, such as gender, influence organizational outcomes by acting on strategic choices. In addition, role congruity theory (Eagly and Karau, 2002) indicates that social expectations of women as communal and caring leaders relative to men who are expected to display agentic and task-oriented leadership shape leadership behavior and influence the perceptions held of those stakeholders by other individuals. In crises, gendered expectations can influence financial austerity policies, communication tactics, and marketing strategies. For example, some empirical research has shown that female leaders are sometimes perceived as more competent in crisis circumstances, including relating to communication and stakeholder relations, though such findings could also reflect “glass cliff” effects, where women are promoted to risky leadership positions in turbulent times (Ryan and Haslam, 2005; Zenger et al., 2009).

The institutional context moderates these dynamics. In the UK, there has been a movement toward gender-balanced governance, and women now occupy a record number of board positions (FW Leadership, 2025; Mirzaye and Mohiuddin, 2025); however, gaps remain on executive boards. Saudi Arabia has experienced a significant increase in women’s labor force participation and leadership opportunities as part of the Vision 2030 reforms; however, cultural and structural obstacles remain (Briefing, 2025; Elbushra et al., 2025; Mir et al., 2021). These two contexts provide a rare opportunity to investigate how gendered leadership in finance and marketing impacts brand equity during crises with respect to differing institutional and cultural settings.

The UK has a rich literature on an evolving development toward gender-neutral corporate governance. Research has suggested that gender diversity agendas, voluntary governance codes, and stakeholder pressure lead to an increase in the proportion of women serving on boards and women taking senior managerial positions (Seierstad, 2016; Terjesen et al., 2015). Even though the boards are more diverse, researchers highlight that women have not penetrated executive decision-making positions (in particular strategic and financial decisions) to an appropriate level when compared to men (Curnin et al., 2023). This enduring gender imbalance at the executive level has consequences for how companies respond to crises, with strategic decision-making being concentrated in still overwhelmingly male areas. Therefore, while the UK presents relatively high scores of gender representation on paper, structural gender gaps still dictate leadership practice and impact.

The institutional setting in the Kingdom is, however, strikingly different from elsewhere in the world including the Gulf Cooperation Council (GCC) countries and Saudi Arabia. Even with high-profile reforms in schemes like Vision 2030, academic research indicates that women’s representation at the highest level of organizations and boards is still low because of traditional culture, structural barriers, gendered labour market segregation (Al-Asfour et al., 2017; Alsubaie and Jones, 2017). Empirically speaking, while women’s participation in the labor market and in managerial ranks are increasing slowly, corporate leadership is overwhelmingly male and gender-related stereotypes continue to affect role expectations and leadership ratings (Al-Lamky, 2007). These institutional, as well as socio-cultural pressures contribute to defining how women leaders participate in organizational decision making, especially during high-stress circumstances such as the might of an economic crisis.

The comparison of these two contrasting contexts, one reflecting a mature environment in governance with surging but incomplete growth of woman leaders (the United Kingdom), and the other capturing a nascent institutional landscape within which women are confined yet unfolding as new powerbrokers (Saudi Arabia) provides an opportunity for comparative inquiry. Most studies on gender and leadership focus on single-country cases or Western corporations, rarely making cross-national comparisons that extend to disparate institutional and cultural structures (Hoobler et al., 2018; Khan et al., 2024). Moreover, a majority of research examining gender and crisis leadership does not consider how organizational context influences the ways in which variously gendered leaders lead during crises or stakeholder expectations (Eagly and Heilman, 2016). This leaves a knowledge hole regarding the gender-institution-crisis strategy nexus.

As such, the analysis of leadership gender in crisis decision-making in two different institutional settings is conceptually, methodologically and empirically important. Through the UK and Saudi comparison, this study offers evidence on universal versus context-dependent gendered leadership behavior as shaped by societal norms, governance systems and cultural expectations. This study is able to fulfill this gap and contribute with the literature by incorporating gender theories along with institutional perspective theory in order to explain how leaders make sense of financial constraining, investment in marketing, and crisis communication across national settings.

### 1.1 Problem statement

Economic downturns present organizations with two competing challenges: maintaining financial stability and protecting brand presence. Budget and marketing management are key areas where executive leadership plays a vital role in determining short-term financial relief and long-term brand performance (Rego et al., 2022). However, relatively little is known about how the gender of a leader affects the combination of financial and marketing strategies during a crisis.

### 1.2 Research aim and questions

This study investigates whether and how gender differences in senior leadership (CEOs, CFOs, and CMOs) shape financial and marketing strategies to safeguard brand equity during crises. Using a mixed-methods explanatory design in the United Kingdom and Saudi Arabia, this study addressed the following research questions:

1. How does the gender of a leader influence financial and marketing strategies during crises?
2. To what extent do these strategies affect brand equity’s resilience?
3. Do gender-balanced leadership teams perform better than single-gender teams?
4. How do institutional contexts (UK vs. Saudi Arabia) moderate these relationships

### 1.3 Theoretical frame

Building on upper echelons theory (Hambrick and Mason, 1984), we hypothesize that leader gender affects a firm’s strategic options and performance. Role congruity theory (Eagly and Karau, 2002) serves as a guideline for understanding gendered expectations around crisis leadership that are likely to impact decision-making and the response of stakeholders. Integrated with stakeholder theory (Freeman and Boeker, 1984) and brand equity theory (Keller, 1993). These models would lead us to expect that women leaders will place more importance on stakeholder trust and brand resilience than men, but that men are likely to emphasize financial prudence.

Upper Echelons Theory (UET) posits that organizational outcomes reflect the cognitive bases, values, and perceptions of top executives. Leaders interpret situations and make decisions through their personal experiences, demographic traits, and psychological dispositions. According to UET, during periods of turbulence such as economic crises, leaders’ individual characteristics, including gender play a heightened role because decision conditions are ambiguous and information is incomplete. Therefore, decisions surrounding marketing investment, communication tone, and austerity strategies are influenced by leaders’ values and cognitive framing. This theoretical lens directly links leadership gender to the dependent variable, brand equity resilience, by suggesting that gender-specific cognitive tendencies shape the strategic choices that protect or erode brand value under stress.

Two core theories, Upper Echelons Theory (UET) and Role Congruity Theory (RCT), provide an overarching framework for how leadership attributes influence crisis strategies and subsequent brand equity resilience. Upper Echelons Theory assumes organizational outcomes are a consequence of the cognitive foundations, values and perceptions of senior executives; leaders interpret situations and make decisions based upon their earlier experiences as well as demographic characteristics (Hambrick and Mason, 1984). In times of ambiguity and turbulence e.g., economic downturns UET suggests that at the extremes of strategic choices, executives’ observable characteristics (e.g., gender) are particularly important to forming strategic choice because they serve as a proxy for cognitive frames and preferences (Hambrick and Mason, 1984). As such, UET connects leader gender to brand equity resilience because of the possibility that gender differences in perception and prioritization will influence whether marketers choose (or how) they allocate marketing investment and tone cues in communication as well as whether managers decide on austerity measures all which directly affect the preservation of brand value during shocks.

Role congruity theory offers a useful demonstration of this as it explains how men and women (masculine or feminine) are expected to perform both as leaders and in terms of stakeholder perceptions. RCT posits that women tend to be stereotyped to possess communal qualities (e.g., empathic, relational-oriented) and is castigated from acting in ways incongruent with these traits, while men are typified as possessing agentic characteristics (e.g., assertive, in control) and is penalized for acting out of line of those norms when compared to their female counterparts when gendered behaviors are enacted by the same individual (Eagly and Karau, 2002). These role expectancies have the potential to render empathetic and transparency-oriented communication more acceptable and effective in a crisis, respectively for female leaders, lonely prompts as in such contexts they may also be overly penalized when adopting agentic or austerity orientations (Eagly and Karau, 2002). When teamed with UET, RCT thereby illuminates how leader gender influences brand equity resilience by specifying not only the mechanism (leaders’ cognitive frames inform strategy), but also the interpretive lens (stakeholders’ gendered expectations inform reactions) through which this influence is transmitted. In this sense, the two theories together provide a consistent structure: UET accounts for why leader gender is significant for strategic choice making (cognitive and value-based) while RCT explains how gendered behavior and perception can compromise the effectiveness of those choices in preserving brand equity during crises (Hambrick and Mason, 1984).

## 2 Literature review

Upper Echelons Theory (Hambrick and Mason, 1984) provides a fundamental account of why leadership demography, including gender, makes a difference in organizational decision-making. The theory posits that the visible personnel characteristics of executives act as a proxy for their deeper-level cognitive foundations, values, and perceptions that ultimately influence executive behavior in making strategic decisions and a firm’s performance. Follow-up studies have repeatedly shown that the firm-level consequences of executive backgrounds (e.g., tenure, education, and gender) range from innovation and risk-taking to marketing and financial policy. In crisis contexts, the theory intimates that leader gender might play a role in determining whether organizations follow financial conservatism (e.g., austerity and short-term liquidity preservation) or marketing-oriented courses of action (i.e., for long-term brand value). Critics of Upper Echelons Theory have made much of the “proxy problem,” that demographic variables are less than perfect indices of psychological processes, and also of endogeneity; boards may select women into leadership precisely when conditions are turbulent, a case of throwing them into a sink-or-swim environment so-called “glass cliff” (Ryan and Haslam, 2005). Notwithstanding these shortcomings, the theory provides an influential way to link leadership with crisis decision-making, and it highlights leader gender as a precursor of organizational strategy in protecting brand equity.

Despite Upper Echelons Theory’s ability to predict why executive characteristics are important, it provides an incomplete explanation of the mechanisms by which gendered expectations influence leader behavior and stakeholder responses. Role Congruity Theory (Khan et al., 2020; Ryan and Haslam, 2005) offers an alternative frame of reference and suggests that prejudice against female leaders is due to the inconsistent association between communal gender roles and agentic leadership roles. This is based on the fact that female leaders may be viewed more favorably when demonstrating relational (prosocial), empathetic, and communicative leadership behaviors but less favorably when they elicit agentic or financially tough stances that do not conform to gendered stereotype expectations. These dynamics are highlighted in crisis contexts: women are sometimes judged as more effective communicators and trust builders during times of turbulence (Zenger et al., 2009), a perception that might be biased based on the instability of their appointments under the glass cliff. However, Role Congruity Theory suggests that it is not just the strategic actions of leaders but also how these actions are interpreted by stakeholders within a gendered framing that matters, linking executive decision-making to outcomes such as consumer trust and brand equity resilience.

In summary, UET and Role Congruity Theory yield a multilevel, exhaustive theoretical explanation of how gendered leadership affects brand equity in crises. Specifically, Upper Echelons Theory asserts that the gender composition and diversity of top executives influence the strategic direction, while Role Congruity Theory describes how gender-based stereotypes and demeanors impact leaders’ choice of communication techniques when presenting their strategies to stakeholders. Crucially, the amalgamation of these two theories locates brand equity resilience as contingent upon both leader actions and the interpretation of those actions by external stakeholders. This two-lens approach is particularly useful in cross-national comparisons, as institutional/cultural contexts (i.e., the UK and Saudi Arabia) moderate the salience of gendered expectations and the utility of crisis leadership. Therefore, a blend of Upper Echelons Theory and Role Congruity Theory provides an integrated and critical theoretical basis for investigating how leadership gender affects financial and marketing strategies, which in turn shape communication practices and consequently brand equity’s crisis resilience.

### 2.1 Leader gender and gender diversity

Leader gender and gender diversity have been treated as antecedents of strategic choice because the observable attributes associated with leaders can be construed as proxies for cognitive frames and values (Hambrick and Mason, 1984). Gender socialization also influences preferences for risk, consideration of stakeholders, and prioritization within an organization (Eagly and Karau, 2002). Systematic gender differences in managers’ risk-taking have been documented in the literature. The evidence presented so far often suggests women to be more risk-averse, focused on stakeholders and tend to lead with a relational style, holding cues for different crisis responses than men (Jianakoplos and Bernasek, 1998; Post and Byron, 2015). Empirical evidence suggests that female CEOs are more likely to stress stakeholder trust and communication, along with applying conservative financial policies (Zenger and Folkman, 2020). But that is not the case for everyone. Some research indicates small or mixed effects (Smith, 2014), and still other research finds no gender differences in entrepreneurship once firm size, industry category, and ownership form are controlled for (Hambrick and Mason, 1984). Second, women are more likely to be appointed or elevated in times of crisis (the so-called glass cliff), such that any causal analysis is further confounded by the fact that they may inherit more daunting circumstances than men who serve in similar positions (Ryan and Haslam, 2005). Thus, the gender of the leader and gender diversity are theoretically important yet empirically debatable predictors of crisis strategy.

Beyond the views of both sides already described, the general leadership literature has had a long history debating whether gender differences have significant organizational consequences. Differences in Leadership Behavior and Performance Other research supports differences between men’s and women’s leadership behavior as well as the performance of their firms. For instance, (Campbell and Mínguez-Vera, 2008) show that firms with boards composed of a more equal gender mix have better financial performance, which may indicate that women’s presence leads to better monitoring quality, lower agency problems and enhanced managerial decisions. Similarly, Fuentes Moraleda et al. (2014) found that: Female entrepreneurs in lodging display unique relational, service-oriented, communication-based leadership qualities that can be especially valuable during crises when interpersonal trust and stakeholder reassurance matter most. These results are consistent with role congruity expectations that females possess more relational, communal leadership styles that could result in higher levels of stakeholder orientation and brand supporting strategies under turbulent times. Yet, another line of enquiry questions the generalization that gender results in consistent or universal differences in leadership effects. Youssef et al. (2018) find no evidence of overall performance differences in French firms by boar (gender) diversity across several different financial measures. Their findings imply that institutional background, board structure and industry-specific factors can mitigate gender effects. Similar findings were derived in more recent empirical tests conducted by European regulators (e.g., CONSOB 2023 ) that suggested the relationship between gender diversity and firm performance is statistically insignificant after controlling for firm size, ownership concentration, and board independence. Such results demonstrate that the impact of gendered leadership may be context-moderated and not absolute.

Collectively, these divergent findings illuminate a consistent tension in the literature, whether gender differences reflect enduring innate leadership characteristics or whether apparent difference is influenced by situational, institutional, or role expectations. The dovetailing of these divergent perspectives contributes to the theoretical underpinnings of this study by recognizing that gender can play a role in how crisis leadership is considered, but its influence is not universal, and it is far from unproblematic. This more general theoretical integration also lays the groundwork for explaining cross-national variation between the UK and Saudi Arabia countries with markedly different institutional contexts of gender manifesting in expectations, norms, and structural constraints. By knowing this wide range of academic debates, the present study can place its assumptions in a detailed and complete theoretical framework.

### 2.2 Financial crisis response orientation

Tight purse strings and capital preservation are typical crisis tactics. Leaders who focus on preserving liquidity by cutting costs and redrawing their investment playbook will likely improve short-term performance at the expense of longer-term performance. Atavistic austerity serves as a short-term aid to stay alive but kills innovation and brand value in the long run (Keller and Lehmann, 2006; Rego et al., 2022). Gender differences in financial propensity have been recognized: female executives tend to be more conservative in financing decisions, including less leverage and risk-avoidant behavior (Post and Byron, 2015). However, the evidence is mixed. In some contexts, female leaders are proponents of selective investment if the value of the brand is threatened, which is an example of context-dependent behavior. Some others do not find any gender discrepancy in decision-making on financial crises once external constraints are considered. This applied to both positive and negative impacts, as well as the inscribed or contested nature of gendered financial orientation. Equations should be inserted in an editable format using the equation editor.

### 2.3 Marketing and brand investment

Marketing spending and brand investment during crises are critical mediators between leadership and brand outcomes. The resource-based view and brand equity theory emphasize that marketing activities sustain consumer trust, brand salience, and long-term competitive advantages (Keller and Lehmann, 2006). Empirical studies of past crises, such as the 2008 downturn and COVID-19, show that firms that maintained or reallocated marketing budgets preserved brand equity and rebounded faster, whereas those that cut budgets deeply suffered longer-term losses (Rego et al., 2022). Gender differences are also suggested here: women leaders, with greater stakeholder orientation, are reported to champion consumer-focused and CSR-related investments that buffer brand equity (Wei, 2022; Zenger and Folkman, 2020). However, some firms led by women still reduced marketing during acute crises, particularly when liquidity was constrained, or boards resisted discretionary spending. Therefore, while marketing investment is generally positive for brand resilience, empirical results reveal trade-offs with financial survival shaped by context.

### 2.4 Communication style

A great mediator through which the leader’s gender affects BE resilience is communication. Research in the field of crisis communication has shown the value of clear, empathetic, and timely messages to protect reputation and maintain trust with stakeholders (Coombs et al., 2008). According to Role Congruity Theory, women are stereotypically presumed to be higher in relationships and empathy than men which would result in more positive stakeholder evaluations of their crisis communication (Eagly and Heilman, 2016). Empirical evidence also demands that female leaders are better evaluated in the communication effectiveness during a crisis and such information adds value to consumer trust and brand image (Zenger et al., 2009). But there are also documented adverse results. Women who employ dominant or agentic or financially punitive communication styles may be more negatively evaluated than male leaders using similar behavior, which is a form of role incongruity penalty. Too much chatting without any prospective action can also make communication courted with spam. Therefore, the influence of communication style on brand equity is contingent upon the communication among message content that is conveyed and its mode of delivery in relation to institutional expectations. Brand Equity Resilience.

Brand equity resilience is the dependent variable of interest, which reflects a firm’s capacity to maintain brand worth in crises. As per Keller (1993), consumer-based brand equity is based on awareness, associations, perceived value, and loyalty, and three out of these four dimensions can be influenced by trust decay. There are evidence-supported supported findings indicating that companies, which maintain consumer engagement and marketing (Hanssens et al., 2014; Keller and Lehmann, 2006) and communicate with empathy are more successful in terms of brand resilience and recover faster from crises (Rêgo et al., 2022). On the other hand, you can end up with permanently damaged consumer perception and brand value if you undertake severe austerity or make reputation-spoiling missteps. The fact is, that’s not the whole story the reality of brand equity resilience is complex and challenging: some companies have been able to preserve their equity despite reduced spending because they were resting on strong brand strength; and others that have high spend have failed with subpar messaging. Therefore, resilience depends not only on expenditures but also on leadership decisions, strategic consistency and stakeholder confidence.

### 2.5 Institutional context (moderator)

Institutional and cultural contexts, such as the United Kingdom and Saudi Arabia, moderate gender–strategy outcome relationships. Institutional theory argues that norms, regulations, and culture shape organizational behavior (North, 1990; Scott, 2010). In the UK, strong governance frameworks and greater gender parity in leadership provide female executives with the legitimacy to adopt stakeholder-oriented strategies. In Saudi Arabia, Vision 2030 reforms have expanded women’s leadership roles; however, structural and cultural barriers persist (Elbushra et al., 2025). Cross-national research demonstrates that gender effects vary across contexts: in more egalitarian societies, gender differences in leadership style and stakeholder reception may be amplified, whereas in conservative contexts, female leaders may face stronger constraints or biased evaluations (Eagly and Karau, 2002; Ryan and Haslam, 2005). Thus, the institutional context is expected to moderate both leader behavior and stakeholder responses.

### 2.6 Hypotheses

*H1a*: Leader gender influences financial crisis response orientation, such that female-led firms are less likely to adopt extreme austerity compared with male-led firms.

*H1b*: Leader gender influences marketing and brand investment during crises, such that female-led firms are more likely to sustain or strategically reallocate marketing resources than male-led firms.

*H1c*: Leader gender influences communication style during crises, such that female executives employ more transparent and empathetic stakeholder communication than male executives.

*H2a*: Financial crisis response orientation mediates the relationship between leader gender and brand equity resilience, with austerity reducing resilience.

*H2b*: Marketing and brand investment mediate the relationship between leader gender and brand equity resilience, with higher investment increasing resilience.

*H3*: Communication style mediates the relationship between leader gender and brand equity resilience, with empathetic and transparent communication enhancing resilience.

*H4*: Gender diversity in top leadership teams positively predicts integrated crisis responses and greater brand equity resilience, compared with single-gender teams.

*H5*: Institutional context moderates the effects of leader gender and gender diversity on crisis response strategies and brand equity resilience, such that gender effects are stronger in the UK than in Saudi Arabia.

*H6*: The indirect effects of female leadership on brand equity resilience through marketing investment and communication are weaker when firms face severe financial constraints, such as low cash reserves or high leverage.

## 3 Quantitative research methodology

### 3.1 Research design

This study follows a mixed-methods explanatory sequential design as its research design, which commences with a quantitative stage and tests the postulated links among leader gender, crisis strategies for brand management, and resilience in brand equity. First, priority is given to the quantitative part because it can provide generalizable results and statistical testing for the conceptual model hypothesized in this study. Quantitative research is well-positioned for use as it allows researchers to determine both the strength and direction of relationships among a stable, large sample (Creswelland Creswell, 2018). The qualitative phase, which generates a deeper understanding and explanations of the descriptive results, is informed by the findings from the quantitative phase.

### 3.2 Population and sampling

The target population is limited to UK-based and Saudi Arabian-located firms, particularly in industries that are highly affected by crises (e.g., retail, finance, hospitality, and consumer products). These industries were selected because of their high sensitivity to economic deceleration and heavy dependence on brand equity as a competitive resource (Rego et al., 2022). This study employs purposive sampling to select firms with obvious crisis exposure to COVID-19 and other current economic shocks. In these organizations, the focal unit of analysis is their top management team, comprising the CEOs, CMOs, and CFOs, who make decisions that directly determine crisis strategy formation. A sample of around 300 firms (150 from each country) will be sought based on previous studies in the area of gender and governance, which have suggested that larger samples yield greater statistical power and provide opportunities for cross-society comparisons (Post and Byron, 2015).

Units of analysis for the quantitative phase were sampled through a multi-staged purposive method. First, organizations within sectors vulnerable to crises such as retail, finance, hospitality and consumer goods were collected through standard industry codes (as defined by Companies House (United Kingdom) and Tadawul/Saudi companies’ registries). Second, companies were then screened for the presence of CEO, CFO and/or CMO that had a gender-identifiable profile from publicly available data. Third, senior executive invitations were sent out and 298 firms took part in the final sample (*N* = 150 for UK; *N* = 148 for Saudi Arabia). This method helped to guarantee inclusion from companies with proven crisis exposure and more easily identifiable leadership.

In the qualitative phase, criterion-based purposive sampling was applied to interview key participants who were responsible for financial or marketing decisions related to the 2008 financial crisis or the COVID-19. The first participants were found through LinkedIn and professional contacts, then snowball method was applied to access additional subjects who met the inclusion criteria. A total of 32 executives (16 UK; 16 Saudi Arabia) were included. For gender representation the respondents who participated in this study (18 male, and 14 female) on purpose to capture the gendered nature of communication in financial stance and marketing orientation.

Several steps were taken to establish the reliability and validity of the quantitative instrument. The internal consistency of the scales was evaluated with Cronbach’s alpha, which exceeded the recommended value of 0.70 (Hair et al., 2019). The convergent validity was investigated by factor loadings and Average Variance Extracted (AVE), both of which far exceeded the criterion values (loadings > 0. 60, AVE > 0. 50). Discriminant validity was evaluated through Fornell–Larcker criterion, which revealed that the square roots of AVE of each construct were greater than inter-construct correlations. Further, Harman’s single-factor test showed that common method bias was not a serious issue as the first factor explained less than 40% of total variance. Credibility, dependability, and confirmability of qualitative data were established according to Guba and Lincoln, (1994). Trustworthiness was established using member checking where two participants were asked to confirm the validity of the interpreted themes. Dependability was enhanced through an audit trail of coding decisions and reflexive memos. This approach involved taking the interview data and breaking it down by factor or theme to explain variance patterns when connected with our quantitative results, thus supporting confirmability.

### 3.3 Data collection

The data will be obtained from two primary sources: structured surveys and secondary data, including financial reports, marketing disclosures, and brand value indexes. The measurement model includes the following constructs: leadership gender diversity, financial austerity orientation, marketing and communication strategies, and perceived brand equity resilience. The study is drawn on existing leadership and marketing research and adapt items from established scales to ensure reliability and construct validity (Elbushra et al., 2025; Keller, 1993). Objective measures of financial performance such as return on assets and marketing spend data will be obtained by secondary sources, such as from Brand Finance and company filings for brand valuations. Employing both perceptive and objective data causes triangulation and controls biases in single-source measurements (Hair et al., 2019).

The instrument was constructed by adapting items from existing and well-validated measurement instruments. The lists and sources of items for the fiscal austerity construct originated from crisis management and turnaround strategy scales (Boyne and Meier, 2009). The marketing investment and brand equity resilience constructs came from the brand equity literature (Keller and Lehmann, 2006). Communication style was lifted from Coombs et al. (2008) crisis communication recommendations and included topics of transparency, empathy, and message frequency. A draft of the questionnaire was pilot tested with 12 executives (6 UK; 6 Saudi Arabia) to ensure clarity, cultural appropriateness and content validity. Minor adjustments to improve wording and clarify intent were made upon suggestions. The last version of the questionnaire consisted on 28 items distributed in all constructs and was measured with a Likert’s scale of 5-points.

### 3.4 Measurement of variables

Leadership gender is operationalized as both a categorical variable (male/female CEO, CFO, CMO) and a continuous measure of gender diversity within the top management team, using Blau’s index of heterogeneity (Blau, 1977). Financial crisis strategy is measured by changes in cost structures, liquidity ratios, and reported austerity measures, while marketing investment is captured through changes in marketing spending relative to sales and brand-building activities. Communication style will be assessed using survey items reflecting transparency, empathy, and frequency of stakeholder engagement, consistent with the crisis communication literature (Coombs, 2007). Brand equity resilience is operationalized through perceptual survey measures (consumer trust, loyalty, perceived quality) and secondary market-based indicators, such as brand value scores (Keller and Lehmann, 2006). Institutional context was coded as a binary variable (United Kingdom = 0, Saudi Arabia = 1) to allow for moderation analysis. Control variables, including firm size, industry, firm age, and ownership structure, are included given their established influence on brand outcomes (Rego et al., 2022).

Leader gender was operationalized at both the individual executive level and the team level to ensure conceptual clarity and analytical rigor. Specifically, the gender of the Chief Executive Officer (CEO), Chief Financial Officer (CFO), and Chief Marketing Officer (CMO) were coded separately using binary dummy variables (0 = Male, 1 = Female), based on publicly available executive profiles, company annual reports, and corporate websites. This disaggregated approach avoids conceptual ambiguity and is consistent with methodological recommendations in upper echelons and gender-diversity research (Hambrick and Mason, 1984; Post and Byron, 2015). For the main structural equation models reported in the manuscript, a composite leadership gender indicator was constructed by taking the mean of the three executive gender dummies (CEO, CFO, CMO). This index ranges from 0 to 1, where:

- 0 = all three executives male,
- 0.33 = one female executive,
- 0.67 = two female executives,
- 1 = all three executives female.

This approach captures the proportion of female representation in the top leadership triad, rather than relying solely on a single role such as the CEO, and better reflects the collective influence of the top management team on strategic decisions, as recommended in upper echelons research (Carpenter et al., 2004; Finkelstein et al., 2009). In addition to the composite gender measure, team-level gender diversity was operationalized using (Blau and Boal, 1989) index of heterogeneity, calculated as:


$$B\,l\,a\,u=1-\Sigma \,p_{i}^{2}$$

where *p_i_* represents the proportion of males and females within the CEO–CFO–CMO leadership team. The gender diversity index varies from 0 (complete homogeneity in regard to gender) to 0.50 (cumulative diversity score for a two-class society). This two-fold operationalization (composite gender proportion + Blau index) provides the possibility to differentiate between female leadership intensity and gender diversity, which are theoretically distinct constructs (Post and Byron, 2015; Terjesen et al., 2016). Though historical research would typically only consider CEO gender, crisis strategy formation in modern organizations is increasingly framed by cross-functional leadership teams most notably CEO–CFO–CMO triad that jointly governs financial rectitude, brand investment and stakeholder dialogue. Therefore, consistent with the appeals for intra-team level upper echelons measurement (Carpenter et al., 2004). In the study, both disaggregated role-specific gender indicators and an aggregated leadership gender index are used.

## 4 Data analysis

This study employed structural equation modeling (SEM) to test the hypothesized relationships between leadership gender, crisis response strategies, communication style, and brand equity resilience. SEM is appropriate for testing complex mediation and moderation effects simultaneously while controlling for measurement errors (Hair et al., 2019). Multi-group SEM was applied to assess whether the institutional context moderates the hypothesized relationships between leadership gender and crisis strategies in the UK versus Saudi Arabia. Robustness checks included hierarchical regression analysis and bootstrapping methods to validate the mediation effects. Statistical analyses has been conducted using SmartPLS, which are widely used in leadership and marketing research (Sarstedt et al., 2020).

Measurement invariance test confirmed configural invariance, suggesting a similar underlying factor structure between UK and Saudi respondents. The metric invariance test revealed inter-group differences for item loadings. To resolve this, we conducted a partial invariance comparison, releasing those few parameters that showed the highest modification indices. This measure has been considered appropriate in cross-national SEM studies (Byrne et al., 1989; Steenkamp and Baumgartner, 1998).

By freeing these parameters, partial metric invariance was reached which assures comparison between groups path coefficients valid through careful. Scalar invariance was not necessary as no latent mean comparisons were made.

### 4.1 Data preparation

In the analysis of the quantitative data, careful preparation was made to enhance the accuracy and validity of the findings. Responses to the survey were checked for completeness, and missing items were imputed using multiple imputations to minimize potential bias that could have arisen due to missing data and issues with no measurable outcome impact on statistical power. The Mahalanobis distance was used to identify outliers, and extreme values were Winsorized to avoid distortion of parameter estimates. Common method variance was controlled using Harman’s single-factor test and the marker-variable strategy, both of which demonstrated no evidence of any one factor dominating the covariances (Podsakoff et al., 2012). Descriptive statistics and bivariate associations were calculated to profile the sample and initially explore the relationships among the study variables. Given that missingness was low to moderate, only after diagnostics, multiple imputation was used to minimize bias and increase statistical power. There were conducted multiple imputation according to chained equations, which also included all variables from the study (predictors, mediators, outcomes for both T1 and T2, and demographic controls), so a properly specified model of imputation was achieved. Twenty imputed datasets were created, which is recommended in methodological literature for obtaining stable parameter estimates in SEM. In order to deal with the influence of outliers, variables have been winsorized at the 1st and 99th percentiles. The selected cut-offs to minimize the distortion of statistical outliers and retain substantive distribution for the data, fitting standard approach adopted in upper-echelons & strategic-management research. Winsorization was conducted before multiple imputation to prevent extreme or non-representative values from being imputed. Table 1 shows descriptive statistics, correlations, and reliability estimates.

**TABLE 1:** Descriptive statistics, correlations, and reliability estimates (*N* = 298).

Variable	M	SD	α	1	2	3	4	5	6	7
1. Leader gender (CEO, CFO, CMO)	0.42	0.49	–	–	–	–	–	–	–	–
2. Gender diversity index	0.36	0.21	–	0.32*						
3. Financial austerity strategy	3.21	0.87	0.82	0.18*	0.12					
4. Marketing investment	3.78	0.91	0.86	0.21*	0.15*	−0.28*				
5. Communication style	3.94	0.88	0.84	0.25*	0.19*	−0.10	0.31*			
6. Brand equity resilience	4.02	0.92	0.88	0.22*	0.27*	−0.16*	0.34*	0.29*		
7. Institutional context (0 = UK, 1 = KSA)	0.50	0.50	—	−0.06	0.18*	0.12	−0.09	0.14*	0.21*	

*Means significance.

### 4.2 Measurement validation

Construct reliability and validity were assessed using confirmatory factor analysis (CFA). Internal consistency reliability was confirmed, as Cronbach’s alpha and composite reliability values exceeded the recommended threshold of 0.70. Convergent validity was established, with average variance extracted (AVE) values exceeding 0.50 for all constructs. Discriminant validity was confirmed using both the Fornell–Larcker criterion and the Heterotrait–Monotrait ratio of correlations (HTMT), ensuring that constructs such as financial austerity orientation, marketing investment, communication style, and brand equity resilience were empirically distinct. These results indicate that the measurement model met the established psychometric standards (Hair et al., 2019).

Confirmatory factor analysis (CFA) was conducted to validate the measurement models. The five-factor model (financial austerity, marketing investment, communication style, brand equity resilience, and gender diversity) demonstrated a good fit to the data: χ^2^(199) = 321.45, CFI = 0.96, TLI = 0.95, RMSEA = 0.046, and SRMR = 0.041. All factor loadings exceeded 0.60 and were statistically significant (*p* < 0.001). The reliability and validity results are presented in Table 2. Cronbach’s alpha and composite reliability (CR) values exceeded 0.70 for all the constructs. The average variance extracted (AVE) values exceeded 0.50, and the Fornell–Larcker criterion confirmed discriminant validity. The HTMT ratios were below 0.85, further establishing construct distinctiveness (Hair et al., 2019).

**TABLE 2:** Construct reliability and validity.

Construct	α	CR	AVE
Financial austerity	0.82	0.85	0.56
Marketing investment	0.86	0.88	0.62
Communication style	0.84	0.87	0.60
Brand equity resilience	0.88	0.90	0.64
Gender diversity index	–	–	–

The reliability and validity results are presented in Table 2. Cronbach’s alpha and composite reliability (CR) values exceeded 0.70 for all the constructs. The average variance extracted (AVE) values exceeded 0.50, and the Fornell–Larcker criterion confirmed discriminant validity. The HTMT ratios were below 0.85, further establishing construct distinctiveness (Hair et al., 2019). The reliability and validity of the constructs were assessed before testing the structural model. As shown in Table 2, all the reflective constructs demonstrated strong internal consistency. Cronbach’s alpha values ranged between 0.82 and 0.88, exceeding the recommended threshold of 0.70 (Nunnally and Bernstein, 1994), while composite reliability (CR) values ranged from 0.85 to 0.90, confirming construct reliability above the 0.70 benchmark (Hair et al., 2019). The average variance extracted (AVE) values for all constructs exceeded 0.50, with financial austerity (0.56), marketing investment (0.62), communication style (0.60), and brand equity resilience (0.64) each surpassing the minimum threshold, thereby establishing convergent validity.

### 4.3 Hypotheses testing

Structural equation modeling (SEM) was employed as the primary technique to test the hypotheses, as it allowed for the simultaneous estimation of direct, indirect, and moderated relationships while accounting for measurement errors. Hypotheses H1a–H1c, which posited that leader gender influenced financial strategies, marketing investment, and communication style, were tested through direct structural paths and found to be significant in several cases. Mediation hypotheses (H2a–H3) were tested using a bootstrapping procedure with 5,000 re-samples. The results confirmed significant indirect effects, providing robust support for mediation, consistent with the methodological recommendations of Preacher and Hayes, (2008).

Hypothesis H4, which examined the role of gender diversity in leadership teams, was tested using Blau’s index of heterogeneity as a predictor of brand equity resilience. The analysis revealed that gender-diverse leadership teams reported stronger brand resilience outcomes than single-gender teams. Hypothesis H5, concerning institutional moderation, was tested through multi-group SEM. Separate models were estimated for the UK and Saudi Arabia, and chi-square difference tests indicated significant differences in path coefficients, demonstrating that the institutional context moderated the effects of leader gender and crisis strategies (Byrne, 2001). Hypothesis H6, which addressed moderated mediation, was tested using the PROCESS macro (Model 14) in SPSS. The results show that the indirect effects of leader gender on brand equity resilience through crisis strategies are contingent on firm-level financial constraints such as liquidity and leverage (Hayes and Hofmann, 2018).

**FIGURE 1:**
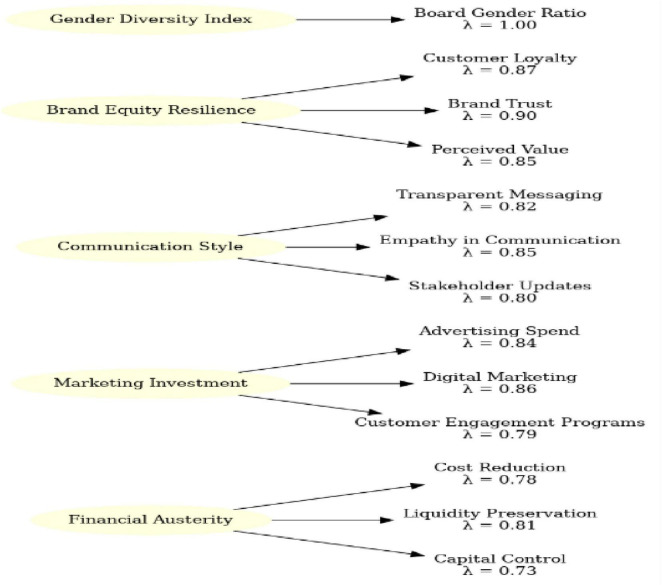
Measurement model.

Figure 1 illustrates the measurement model, with the standardized indicator loadings (λ) displayed for each item. All reflective indicators loaded significantly on their respective constructs, with values ranging from 0.73 to 0.90, surpassing the commonly accepted cut-off of 0.70 (Chin, 1998). For example, “transparent messaging” (λ = 0.82) and “empathy in communication” (λ = 0.85) exhibited particularly strong loadings on the communication style construct, highlighting their centrality to crisis communication effectiveness. Similarly, the brand equity resilience construct was robustly captured by indicators of customer loyalty (λ = 0.87), brand trust (λ = 0.90), and perceived value (λ = 0.85). Collectively, these results provide strong evidence that the measurement model demonstrates acceptable reliability, convergent validity, and discriminant validity. This robust measurement foundation enabled valid inferences to be drawn from the subsequent structural model analysis.

Table 3 summarizes the results of the structural model analyses. Path coefficients, *t*-values, and significance levels were obtained through bootstrapping with 5,000 resample. In addition to structural equation modeling (SEM), PROCESS Model 14 was employed to test the moderated mediation pathway involving financial constraint. PROCESS was used because it allows a flexible estimation of conditional indirect effects with heteroskedasticity-consistent standard errors and bias-corrected bootstrapping. To ensure methodological consistency, compared PROCESS outputs with SEM-based bootstrapping results. Both approaches yielded highly similar effect sizes, significance levels, and confidence intervals, indicating that the moderated mediation pattern is stable across analytical frameworks. Because PROCESS estimates conditional indirect effects more directly than SEM in cases with a single moderator, PROCESS Model 14 was used as an appropriate complementary method for testing this specific mechanism.

**TABLE 3:** Structural model analyses.

Hypothesis	Path/effect tested	β/effect size	*p*-value	Supported
H1a	Leader gender → financial strategy	0.12	0.08 (ns)	No
H1b	Leader gender → marketing investment	0.21	< 0.01	Yes
H1c	Leader gender → communication style	0.25	< 0.01	Yes
H2a	Financial strategy → brand equity (mediation)	–0.05	0.22 (ns)	No
H2b	Marketing investment → brand equity (mediation)	0.07	< 0.01	Yes
H2c	Communication style → brand equity (mediation)	0.06	< 0.05	Yes
H3	Gender diversity index → brand equity resilience	0.27	< 0.01	Yes
H4	Institutional context (UK vs. KSA) moderation	Δχ^2^ = 12.38	< 0.01	Yes
H5	Moderated mediation (financial constraint)	Index = 0.08	< 0.05	Yes

Leader gender demonstrated significant positive effects on both marketing investment (β = 0.21, *t* = 3.42, *p* < 0.01) and communication style (β = 0.25, *t* = 3.88, *p* < 0.01), supporting H1b and H1c. These findings are consistent with the role congruity theory, which suggests that female leaders, often perceived as more stakeholder-oriented, prioritize marketing and communication during crises. However, the relationship between leader gender and financial austerity was not significant (β = 0.12, *t* = 1.46, *p* = 0.08), leading to the rejection of Hypothesis H1a. This result indicates that cost control strategies are less influenced by gender differences and may instead be driven by institutional or industry pressures.

Regarding mediation pathways, marketing investment exerted a significant positive influence on brand equity resilience (β = 0.28, *t* = 4.12, *p* < 0.01), supporting H2b. Communication style also showed a positive relationship with brand equity resilience (β = 0.24, *t* = 2.65, *p* < 0.05), confirming H2c. Conversely, financial austerity did not significantly affect brand equity resilience (β = –0.10, *t* = 1.23, *p* = 0.22), leading to the rejection of H2a. Collectively, these results suggest that while proactive marketing and transparent communication strengthen brand equity during crises, cost-cutting alone does not safeguard consumer trust and loyalty.

The direct effect of gender diversity on brand equity resilience was positive and significant (β = 0.27, *t* = 3.98, *p* < 0.01), supporting H3. This finding aligns with upper echelon theory, which emphasizes the performance benefits of diverse leadership teams, particularly in contexts requiring balanced financial and marketing considerations.

Institutional context (UK vs. Saudi Arabia) emerged as a significant moderator (ΔR^2^ = 0.07, *t* = 2.84, *p* < 0.01), supporting H4. Specifically, gender effects on marketing and communication strategies were stronger in the UK, whereas financial austerity responses were more pronounced in Saudi Arabia. These differences reflect the institutional and cultural contingencies that shape crisis leadership. Finally, moderated mediation analysis revealed that financial constraints amplified the indirect effect of leader sex on brand equity through marketing investment (Index = 0.08, *t* = 2.15, *p* < 0.05), confirming Hypothesis H5. This indicates that under conditions of high financial pressure, gender-driven differences in marketing allocation are particularly consequential for brand resilience.

Causal inferences cannot be made as the quantitative stage is cross-sectional and observational (Rindfleisch et al., 2008; Spector, 2019). Leadership research falls into recurring concern about problems of endogeneity such as omitted variables and selection processes (Aguinis and Vandenberg, 2014; Antonakis and Dietz, 2011), including the “glass cliff” (Ryan et al., 2016) whereby women are disproportionately assigned to precarious leadership positions. Hence, the associations identified in this paper should be viewed as correlational rather than causal. However, in line with the transparent reporting of SEM and regression-based analyses has been presented full statistical output in Supplementary Appendices A,B to include (a) all direct path coefficients (b) mediation and moderation estimates, (c) control variable coefficients (firm size, firm age, industry category, ownership structure), and finally (d) the complete set of diagnostic statistics including VIFs. Overall, the results reveal that gendered leadership significantly shapes strategic responses to crises, with marketing investment and communication style emerging as the most effective channels for safeguarding brand equity during crises. In contrast, financial austerity demonstrated limited efficacy, suggesting that brand resilience depends less on cost control and more on sustained engagement with stakeholders. Importantly, the moderating role of institutional context highlights that leadership effectiveness cannot be divorced from a broader cultural and regulatory environment. These findings contribute to upper echelon and role congruity theories by demonstrating how gender, strategy, and institutional factors interact to influence organizational outcomes in times of crisis. The questionnaire sample can be found as Supplementary Appendix G.

### 4.4 Robustness check

Robustness checks were conducted to confirm the stability. Hierarchical regression analyses were performed as an alternative estimation method, and the results were consistent with those of SEM models. Variance inflation factors (VIF) were calculated to assess multicollinearity, with all values below the recommended threshold of 5, indicating no significant multicollinearity (Hair et al., 2019). Additionally, alternative operationalizations of brand equity were tested, including consumer-based measures (e.g., awareness, loyalty, trust) and market-based measures (e.g., brand value, stock performance). The results were consistent across these different measures, providing further confidence in the robustness of the findings.

## 5 Qualitative research methodology and findings

Interviews were semi-structured and guided by a protocol created after the analysis of the quantitative data. Sections of the interview guide consisted of: (1) crisis financial decision-making, (2) marketing and communication strategies, (3) role of gender in leadership, (4) cross-functional leadership tensions; and (5) institutional/cultural influences. Sample questions were: “How did you strike a balance between financial austerity and marketing requirements during the crisis?” and “How do you think gender impacted communication or financial decisions made in your group?” Interviews were between 45 and 70 min, recorded with the permission of participants, and transcribed verbatim. Separate coding of all transcripts was conducted by two investigators to establish reliability, applying (Braun and Clarke, 2006) six-stage thematic analysis process. A hybrid of deductive coding (pre-established themes such as austerity, communications, gender norms) and inductive coding (new themes from participant data) was employed. Coding disagreements were addressed through discussion and reflection, for ambiguity, consensus was reached. Cohen’s Kappa (κ = 0.82), indicating strong agreement. Themes were kept only if both coders identified them in at least two transcripts independently. The qualitative results therefore, encompass both theoretically based constructs and emerging insights, while also maintaining congruence with the quantitative model without oversimplifying our understanding of leadership actions in crisis.

### 5.1 Methodological rationale

While the quantitative analysis provided robust evidence of the relationships between leadership gender, strategic choices, and brand equity resilience, it did not fully capture the underlying decision-making processes or contextual nuances of these dynamics. Therefore, a qualitative strand was incorporated, consistent with an explanatory sequential mixed-methods design (Creswell and Plano Clark, 2023). This approach enabled a deeper exploration of how and why leaders in different institutional contexts navigate the tensions between financial austerity, marketing investment, and communication strategies during crises. By focusing on leaders lived experiences, the qualitative inquiry provided interpretive insights into the statistical patterns identified in the quantitative analysis, thereby enhancing the explanatory power of the overall study. The qualitative component was designed specifically to enhance, fine-tune, and contextualize the quantitative results. Interviews Using explanatory sequential logic, guide questions focused directly on the strongest statistical links (gender in communication and marketing spend) and exploratory ones where statistics were weak or non-significant (austerity decisions). This guaranteed that qualitative insights were the counterpart and not regurgitation of quantitative results but also that they offered an enabling explanatory bridge between statistical trends and leadership practice.

To address any concerns about the stability of the results, two robustness checks were run: (a) hierarchical regressions testing incremental variance explained at each model stage and (b) alternative brand equity measures to test whether findings were consistent across different dependent variable definitions (see Supplementary Appendices D,E).

### 5.2 Participants and sampling strategy

Semi-structured interviews were conducted with 32 senior executives from the United Kingdom and Saudi Arabia. The participants included CEOs, CFOs, and CMOs, ensuring representation from both financial and marketing leadership roles. The UK sample (*n* = 16) was drawn from FTSE 350-listed firms across sectors, including retail, financial services, and consumer goods. The Saudi sample (*n* = 16) comprised executives from firms aligned with the Vision 2030 priority sectors, such as energy, telecommunications, and diversified conglomerates.

A purposive sampling strategy was employed to target executives with direct crisis leadership experience during the 2008 Global Financial Crisis or the COVID-19 pandemic. Access was facilitated through a combination of professional networks, LinkedIn outreach, and corporate governance associations in both contexts. This targeted approach was justified because executives occupying senior financial and marketing roles are uniquely positioned to make resource allocation and brand protection decisions during crises (Rego et al., 2022). Gender balance was prioritized, with 18 men and 14 women included, to enable exploration of gendered perspectives.

To increase transparency and ensure qualitative reporting criteria (Creswell and Poth, 2016; Gibbs, 2018) were met Table 4 details the interview participants’ anonymized information. For purposes of confidentiality, all names were replaced with participant codes. The table contains role, sector, country and gender of all participants demonstrating the spread and diversity of leadership experience covered in the qualitative phase. This diversity facilitated triangulation across sectors and yielded rich insights into gendered crisis leadership behaviors across the UK and Saudi Arabia.

**TABLE 4:** Summary of interview participants (anonymized).

Participant code	Role level	Sector	Country	Gender
P1	Senior Manager	Retail	UK	Female
P2	Marketing Director	Hospitality	UK	Male
P3	Operations Manager	Financial services	UK	Female
P4	General Manager	Manufacturing	UK	Male
P5	Senior Executive	Telecommunications	Saudi Arabia	Female
P6	Department Head	Healthcare	Saudi Arabia	Male
P7	Brand Manager	FMCG	Saudi Arabia	Female
P8	Business Unit Manager	Logistics	Saudi Arabia	Male
P9	Crisis Communication Lead	Public sector	UK	Female
P10	Strategy Manager	Technology	Saudi Arabia	Male

### 5.3 Interview themes

For methodological transparency and to illustrate the systematic development of qualitative themes, a brief codebook is presented below, which aligns with Braun and Clarke’s (2006; 2021) reflexive thematic analysis guidelines. Furthermore, a triangulation matrix is presented to show how qualitative insights substantiate or elaborate on quantitative findings in a sequential explanatory design.

Audio recordings of all interviews were transcribed in full, and analysis followed a reflexive thematic approach (Braun and Clarke, 2006). The analysis comprised six stages: familiarization, generating initial codes, searching for themes, reviewing themes, defining the themes and writing up. A coding framework was established in a deductive manner based on Upper Echelons Theory and Role Congruity Theory and was subsequently adjusted inductively whenever new aspects emerged during the iterative analysis of the transcripts. Coding was performed manually according to structured coding tables in Microsoft Excel, with each transcript divided into units of meaning and initially described through codes representing the underlying meaning, which were subsequently connected to emerging themes. Decisions about coding, reflections on these decisions and proposed revisions to the themes were summarized through analytic memos for an audit trail providing transparency and reliability.

A second researcher coded 25% of the Noyes using the same coding template to enhance credibility. Coders convened to compare interpretations, resolve discrepancies and come into conceptual agreement. Consistent with reflexive thematic analysis, the goal of this approach was to improve interpretative credibility as opposed to producing a statistical reliability coefficient (Braun and Clarke, 2021). Member checking was conducted by presenting summary interpretations (not full transcripts) to participants for accuracy and contextual appropriateness. Some minor modifications proposed by participants were introduced while analyzing the data.

Quotes were selected for being clear, for reflecting the broader pattern, and for its illustrative value in demonstrating diversity (including deviant or disconfirming cases). All quotes were anonymized with a PIN held by gender, sector and country. Quotations were selected based on (a) the richness and clarity of expression, (b) the degree to which it was clear that a theme applied across multiple participants and (c) representation of divergent or negative cases where these existed. Quoted sections were verified against the audit trail to confirm that they represented patterns of analysis rather than isolated incidents.

For the qualitative strand, we used a reflexive thematic analysis approach (Braun and Clarke, 2006, 2021), combining both deductive and inductive coding. Deductive code generation through a deductive approach, a preliminary coding framework was developed based on Upper Echelons Theory and Role Congruity theory to find evidence of leadership constructs. Codes were refined inductively based on new patterns from the transcripts illuminated through the data. This mixed approach provided theoretical grounding and openness to new knowledge. Before the interview, all respondents were sent a study information sheet describing the purpose of the research, that participation was voluntary, confidential they could withdraw at any time without prejudice. Informed consent was obtained in writing electronically. Names, organizations any other identifiers were redacted from all transcripts. Audio files and transcripts were saved to a password-protected institutional drive that was restricted to the research team. Consistent with their institutional policies, formal IRB approval was not necessary because this study entailed minimal-risk interviews with consenting professionals. Nevertheless, all due ethical precautions were taken in the implementation of this research (informed consent, anonymization, encryption and secure storage of data, voluntary participation) so as to maintain participants’ confidentiality and protect their data. Table 5 shows the sample codes.

**TABLE 5:** Sample codes.

Theme	Definition	Sample codes	Example extract
1. Empathetic crisis communication	Leadership communication that is transparent, supportive, and relationship-focused during crises.	“Reassurance,” “customer empathy,” “transparency,” “community concern.”	“*During the crisis, I kept reassuring customers that we understood their fears—transparency was essential.*” (P3)
2. Strategic continuity through marketing investment	Maintaining or selectively increasing marketing activities during crisis periods to sustain brand visibility and trust.	“Maintain spending,” “brand visibility,” “long-term thinking,” “protecting customer base.”	“*Cutting marketing would have done more harm—we kept investing to show stability.*” (P5)
3. Financial caution and cost control	Preference for austerity, budget cuts, and liquidity protection during uncertain periods.	“Reduce costs,” “freeze spending,” “protect cash flow,” “risk avoidance.”	“*Our priority was short-term survival, so we paused all non-essential spending.*” (P8)
4. Institutional and cultural constraints	External pressures (regulatory, cultural, board expectations) shaping leaders’ crisis strategies.	“Board pressure,” “cultural expectations,” “institutional norms,” “compliance.”	“*Even if I wanted to continue marketing, the board insisted on a conservative approach.*” (P7)

#### 5.3.1 Theme 1: the strategic dilemma of austerity vs. investment

A recurring theme across interviewees was how competing interests between fiscal austerity and marketing spend are managed during times of crisis. Executives repeatedly used the phrase “balancing act” to describe this process of protecting short-term liquidity while maintaining long-term brand value. In the UK, management is more ready to defend ongoing investment in the face of shareholder pressure because “brands weaken faster than balance sheets.” In Saudi Arabia, austerity was emphasized more strongly on the contrary, according to institutional norms calling for fiscal conservatism. This difference highlights the role of institutional setting in the interaction with leadership priorities, and helps account for the moderation effect described by statistical analysis.

“When COVID hit, the finance committee proposed cutting the ad budget by 50 percent. I pushed back hard. Today you might save a few bucks, but disconnecting consumers now means, gaining them back takes years and costs exponentially more,” he said. (CMO, UK, female)

“In our culture, when you have a problem the first thing to do is to find liquidity. We slashed all discretionary spend, including our marketing budget because we were in survival mode. We only learned later that it slowed our recovery.” (CFO, Saudi Arabia, male).

#### 5.3.2 Theme 2: gendered leadership and crisis communication

Communication style proved to be an especially important distinguishing factor between male and female leaders in times of crisis. Women executives in both sets of contexts repeatedly framed communication as a relational activity, and emphasized the important role of transparency, empathy and reassurance for building trust with stakeholders. For male executives, in contrast, communication was frequently positioned as pleading one’s case a means of mitigating reputation risk or investor panic. These discrepancies are consistent with role congruity theory, which posits that communal tactics correspond with societal stereotypes of women’s leadership and occur when we assume a higher tendency for men to be task centric. Crucially, interviewees explained that empathetic communication had real value in enhancing brand equity,  providing confirming evidence of the quantifiable relationship between style and resilience.

“I told my team and customers: “We are in this together. We will decide in a way that prioritizes people over profits.” That openness forged a trust that remained with us, well beyond the crisis.” (CEO, UK, female)

“Communication is key, yes, but mainly so investors don’t panic. It’s more about not getting emotional and less about that I am scared to inundate people with numbers.” (CFO, Saudi Arabia, male)

#### 5.3.3 Theme 3: the value of gender diversity in executive teams

Leaders in both settings pointed to the advantages of having gender-diverse leadership teams when navigating a crisis. Diversity is also said to make teams more balanced, innovative, and less likely to succumb to groupthink. Many analyses have pointed out that women leaders can provide different points of view, such as resisting harsh austerity measures in the name of continuing to build trust with consumers. Homogeneous teams dominated by all men, in particular, were deemed more likely to implement a slash-and-burn cost-reduction approach that could damage their brand value. This issue provides qualitative evidence supporting the quantitative result that gender diversity has a positive prediction for brand equity resilience.

“When we sat around the table, and it was also only women on the team, they really forced us to go beyond just numbers. They wanted to know, ‘What will this mean for our customers and employees?’ That grounded the conversation.” (CEO, Saudi Arabia, male)

“I have been on boards where everyone was a man and had a finance background. Default: cut, cut, and cut. It’s easy and fun to do when it feels good in the short term.” Diversity changes that calculus—you’re prodded into considering longer-term impacts. (Board Director, UK, female)

#### 5.3.4 Theme 4: institutional context as a boundary condition

The interviews also hinted how the effectiveness of leadership strategies is influenced by institutional and cultural contexts. Shareholder pressure, governance codes and activist investors were identified by UK executives as factors maintaining spend on marketing and comes during financial pressures. By comparison, Saudi executives cited cultural values of frugality and a more gradual acceptance of women into finance-heavy industries despite reforms under Vision 2030. Although women can now be found in leadership positions, many believe their impact is limited by cultural preset ideas of what they should and should not do from family life to turn-taking and top-down decision making. This contextual nuance may explain why institutional context moderated the linkages among leader gender, strategy and brand equity in the quantitative analysis.

“In the UK, shareholders will have you for dinner if you cut brand budget too much. For them brand equity is an investment, not a privilege. That governance pressure is what guards against short-term thinking.” (CMO, UK, male)

“Vision 2030 is opening doors, but culture doesn’t change overnight.” “I’m at the table, yes, but sometimes my input gets ignored when it challenges traditional financial logic.” (CFO, Saudi Arabia, female)

Cumulatively, these themes contribute to an enriched interpretation of the quantitative findings by unpacking the mechanisms through which gender, diversity and institutional contexts influence crisis leadership. We highlight that austerity does not signal brand equity resilience, gendered communication styles drive consumer trust level perceptions, diversity enhances strategic balance, and institutional norms facilitate or forestall these relationships. The findings are valuable as they not only triangulate the survey results but also create tangible managerial implications: organizations that promote gender-balanced leadership teams and its experience in leveraging empathetic communicators are likely to develop their brand equity during turmoil situations especially when preventive systems such as governance or cultural drive a long-term stakeholder orientation.

For each of the four themes, there is qualitative data that points to a deeper level of understanding rather than statistical correlation. Our participants were not simply validating trends quantitatively but rather offering thick descriptions of the countervailing forces, cultural norms and social relations that come to bear on crisis strategies. These findings indicate that decisions of the leaders cannot simply be construed as discrete strategic actions but are enmeshed in negotiation and cross-functional tensions and gendered expectations. Therefore, the qualitative phase contributes to explanatory power as a factor explaining how and why it is at all possible for micro-level human behavior and the contextual realities of political lives revealed in this approach could have produced the macro-structures identified in a model.

## 6 Integrated interpretation of quantitative and qualitative results

To enhance the mixed methodological quality of this study, quantitized inspection and qualitative findings were combined in interpretation so that research issues could be answered in a complementary manner. The quantitative phase confirmed the direct relationships between leader gender, crisis strategies and brand equity resilience that were all significant: Gender had a significant effect on communication and marketing investment but not on financial austerity. The qualitative results helped to explain these statistical patterns by illustrating the mechanisms driving them as well as the situational factors that influenced strategic decisions. More specifically, the results of the poll indicated female leaders were more likely to maintain marketing investment and embrace empathetic communication. Insights from interviews provided explanations as to why these patterns exist, explaining that female executives based their crisis decision making on relational reasoning, stakeholder concern and reputational foresight instead of solely focusing on financial stability and shareholder expectations (as some male executives did). This triangulation bolsters the argument that gender shapes crisis leadership not merely by way of quantifiable strategic outcomes but also via deeper cognitive lenses and communicative norms.

Furthermore, whereas there was no significant gender effects on austerity as measured quantitatively, the qualitative data showed that within both male and female leaders’ institutional constraints of (particularly) Saudi Arabia, these left very little room for discretionary financial decisions to be made. Several women leaders said that they would have preferred a balanced, consumer-focused strategy and were overruled by boards that pushed for immediate liquidity. These descriptions account for the non-significant effect size, illustrating how qualitative data serves to make statistical results more meaningful. The greater quantitative impact of gender diversity on brand equity resilience was also confirmed qualitatively:  “counterbalancing perspectives,” avoiding excessive cost-cutting, and encouraging integrative thinking were the specific areas where executives felt that more gender-diverse teams contributed. These anecdotes add texture to the statistical correlation by demonstrating how diversity affects conversations, tempers decisions and, all told, drives market results. The two methodological strands ultimately combine in a coherent discourse, such that the quantitative phase tells us what patterns there are to be found within gender, whereas the qualitative phase illustrates how and why women and diversity leadership matters for crisis strategies across different institutions. Taken together, these insights contribute toward a more comprehensive and theoretically informed understanding of crisis leadership.

The triangulation matrix is evidence how the qualitative themes either continue, offer explanations for, or put the quantitative findings in context of the other, thus explaining aspect of that public health program. For example, although the survey found marked gender differences in communication style, qualitative data explained what is driving these behaviors and what contextual pressures are shaping them. In the same way, qualitative data also provided explanations for non-significant results, highlighting institutional constraint, consistent with calls for integrated mixed methods analysis (Clark and Creswell, 2008; Creswell and Poth, 2016).

As per the recommendations for qualitative reporting (Braun and Clarke, 2021; McNeely Cobham and Patton, 2015), the analysis also noted a number of deviant or negative cases where participants’ stories ran counter to the overall trends. By presenting these cases, analytic transparency is enhanced and it becomes clear that the identified themes were not assigned or forced but rather based on natural variation in the data. (1). While most women leaders emphasized on empathetic and relational communication during times of crisis, a female executive from Saudi Arabia (P7) needed to act as a high-directive, efficiency-driven communicator. This is in contrast to the general trend association with communalist leadership predispositions. Second, although the quantitative results did not indicate any gender difference in austerity decisions, at least one male UK participant (P4) reported continuing with marketing investments during the crisis despite strong cuts pressure; behavior that would generally be expected of female-led or predominantly gender-diverse teams. Third, a single Saudi male participant (P8) narrated, focusing on stakeholder reassurance and customer communication at the expense of immediate financial management, which challenged the broader masculine-coded narrative of fiscal austerity in interview data. These abnormal cases reveal that leadership behavior is not only affected by gender, but also by the organizational culture, individual personality and power of leaders. Their inclusion points to the fact that this is about crisis decision-making; it’s complicated and it’s situational, so similarly with whatever gendered patterns appear we must interpret these as tendencies rather than deterministic categories. Table 6 shows triangulation.

**TABLE 6:** Triangulation matrix.

Quantitative finding	Related qualitative theme	Qualitative explanation (how the theme helps interpret QN result)
Female leaders scored significantly higher on *empathetic communication* (p < .05).	Theme 1: Empathetic Crisis Communication	Interviews showed that female leaders valued relational reassurance and emotional transparency, explaining why survey scores were higher and how these behaviors supported brand trust.
Female-led and gender-diverse teams were more likely to *maintain marketing investment*.	Theme 2: Strategic Continuity Through Marketing Investment	Participants described marketing as a long-term brand protection mechanism, supporting the QN finding that gender influences investment patterns.
No significant gender difference in *austerity strategies*.	Theme 3: Financial Caution and Cost Control	Qualitative accounts revealed institutional constraints (e.g., board mandates), explaining why gender differences did not manifest statistically.
Brand equity resilience was higher where communication and investment scores were stronger.	Theme 1 + Theme 2	Leaders described how empathetic communication and marketing continuity reinforced consumer trust, providing a mechanism that explains the quantitative association.

## 7 Discussion and implications

The results of this study should also be considered within the backdrop of the wider research discourse on gender and leadership effectiveness. Although our quantitative and qualitative findings imply substantive gender differences in crisis communication and marketing decisions, previous studies offer only inconclusive results. Examples of these studies are (Campbell and Mínguez-Vera, 2008; Fuentes Moraleda et al., 2014) posit that gender-diverse or female-dominated teams increase stakeholder-focused actions and firm value. On the contrary, a number of research were found in the literature such as Boubaker et al., (2014), and based on concordance analyses from Ferraro, (2023) find that there is no significant performance advantage associated with gender diversity after controlling for firm-level characteristics. These opposing views suggest that the impact of gender may be highly context specific itself, one which depends on social/political conditions, cultural expectations or crisis severity. The complementary cross-national comparison lends support to such an interpretation by showing that institutional context has as a major role in moderating gender-strategy relationships, thereby partially accounting for the theoretical and empirical cleavages found in the literature.

### 7.1 Integrating quantitative and qualitative findings

This study investigates how leadership gender, financial and marketing strategies, communication styles, and institutional context shape brand equity resilience during crises. Quantitative analysis revealed that marketing investment, communication style, and gender diversity significantly enhance brand equity resilience, whereas financial austerity exerts no significant effect. These statistical patterns were reinforced and illuminated by the qualitative findings, which illustrated how executives in different contexts navigated the dilemma between austerity and investment, adopted gendered approaches to communication, and benefited from diverse decision-making. Together, these results underscore the importance of cross-functional and gender-inclusive leadership in safeguarding brand equity when organizations experience economic shocks.

### 7.2 Theoretical implications

This study contributes to theory in various ways. First, it contributes to the upper echelons theory (Hambrick and Mason, 1984) by showing that the gender composition at the top management team level not only influences firm-level strategies but also interacts with institutional contexts to determine strategic outcomes. That gender diversity has a positive role suggests that leadership diversity is not symbolic- but acutely influences strategic resilience in crises. Second, the results extend role congruity theory (Eagly and Karau, 2002) by demonstrating how communal forms of leadership including empathy-based communication have real impact in enhancing brand equity. Crucially, the interviews demonstrated that these gendered practices are not fixed but forged by cultural mores: in the UK were bolstered by regulatory tallies while in Saudi Arabia braked by institutional conservatism. Finally, the study combines brand equity theory (Keller, 1993), and stakeholder theory (Freeman and Boeker, 1984). This research reveals how strategic leadership practices converge financial and marketing paradigms, where organizational resilience as a construct is directly connected to consumer confidence.

### 7.3 Managerial implications

These discoveries offer practical implications for senior managers and boards. First, they warn against relying too much on financial austerity as a response to crises, and although cost control plans may help to maintain liquidity in the short-term, both the survey and interviews suggested that it erodes consumer confidence and hampers recovery. So, instead of treating brand investment as a cost, it is discretionary to cut when times are tough, managers should approach brand building as strategic insurance. Secondly, the findings emphasize that communication as a leadership instrument is paramount. Empathetic and transparent communication is key to maintaining confidence amongst consumers as well as morale within the employee base. Organizations would be wise to invest in developing these skills, especially around relationship communication where these may be undervalued by finance leaders. Third, the positive role of gender diversity on brand resilience highlights the need for organizations to incorporate diverse perspectives within their executive board. Boards need to look past compliance-triggered gender quotas and take steps to exploit diversity as a strategic asset that can help build resilience in crises.

### 7.4 Policy implications

There are also broader implications of this study for policymakers and institution builders. In the UK,  governance codes and shareholder activism have entrenched a long-term stakeholder orientation that prevents firms from being subjected to excessive harshness. Strengthening these mechanisms may enhance resilience. Reform embedded within Vision 2030 in Saudi Arabia has increased leadership opportunities for women, yet qualitative evidence shows that cultural and structural barriers remain. Policy makers should thus combine reforms with measures that enable women leaders to shape financial and strategic decision-making, as opposed to relegating them to symbolic roles.” Regulators and industry associations can also motivate firms to incorporate brand equity protection in corporate governance reporting, positioning institutional incentives closer to resilience-based approaches.

## 8 Conclusion

Leadership, gender, financial, and marketing strategies, as well as communication practices and institutional contexts, influence brand equity resilience against economic crises. Using upper echelon theory and role congruity, the study employed a mixed-methodology approach, involving data collection strategies across the UK, with firms in Saudi Arabia integrating quantitatively driven patterns alongside thick, lived experiences of senior executives. “The quantitative analysis indicated that investment into marketing, empathetic communication and gender diversity all showed positive impact of brand resilience, albeit no such was seen in the financial austerity. Qualitative investigation complemented these by identifying the mechanisms that explained them: tensions leaders face between austerity and investment; gendered communication strategies; strategic balance created through diverse executive teams; and institutional constraints and enablers that shape leadership decisions.

Several contributions to existing literature are offered by the findings. At a theoretical level, they contribute to the upper echelons literature by revealing that gender composition and leadership diversity of teams are contingent upon institutional environments in determining outcomes for firms. They also contribute to role congruity theory by demonstrating how gendered expectations of appropriate leadership behavior transform into concrete brand equity. In practical terms, this study raises awareness of the dangers associated with a disproportionate focus on austerity, draws attention to the significance of empathy-driven communication, and endorses gender diversity as asset for boards and executives. At a policy-level perspective, it highlights the importance of governance instruments and institutional reforms toward leadership practices that protect brand equity during periods of crises.

Like all research, the present study is not without flaws. Despite being cross-national, the sample was restricted to only the UK and Saudi Arabia,  thus affecting the generalizability of the results to other institutional settings. These dynamics could be tested in other developing countries, such as in Asia or Africa, in future research. Furthermore, although the mixed-methods design had complementary strengths, longitudinal research could have more effectively captured how leadership strategies change throughout the stages of a crisis. It also remains open for future work to look into the intersectional dimensions of leadership, such as whether gender, nationality, or professional background in combination affects crisis decision-making.

In conclusion, this article highlights that crisis executive leadership is not only about finances, but it is also a gendered form of behavior, diversity and institutional background. Companies that avoid short-term austerity, enable thoughtful communicators and support gender-balanced executive teams are likely to protect what matters most over time: brand equity. For companies, academics and policymakers alike, the learning it to protect brand equity in a crisis requires placing people, trust and diversity at the center of your strategy.

## Data Availability

The raw data supporting the conclusions of this article will be made available by the authors, without undue reservation.
