# Supplementary material for: Inclusive leadership and financial–marketing decision-making in crises: gender diversity and brand resilience
Source: Front Psychol. 2026 Feb 18;17:1730375. doi: 10.3389/fpsyg.2026.1730375 (PMC12959285; doi:10.3389/fpsyg.2026.1730375)
Supplement: Supplementary file 1 [file Supplementary_file_1.pdf]

Supplementary Materials

Appendices

Appendix A. Mediation Effects (Indirect Paths)

Mediation Path	Effect Size	p-value	Supported
H2a: Financial Strategy → Brand Equity	–.05	.22 (ns)	No
H2b: Marketing Investment → Brand Equity	.07	< .01	Yes
H2c: Communication Style → Brand Equity	.06	< .05	Yes

Appendix B. Moderation & Moderated Mediation

Hypothesis	Effect Tested	Statistic	p-value	Supported
H4	Institutional Context (UK vs KSA) Moderation	$\Delta\chi^2 = 12.38$	< .01	Yes
H5	Moderated Mediation (Financial Constraint)	Index = .08	< .05	Yes

Appendix C. Variance Inflation Factors (VIFs)

Variable	VIF
Leader Gender	1.22
Gender Diversity Index	1.31
Financial Strategy	1.45
Marketing Investment	1.38
Communication Style	1.41
Firm Size (control)	1.52
Firm Age (control)	1.47
Ownership Structure (control)	1.33
Industry Category (controls)	1.10–1.50

Appendix D. Hierarchical Regression Results (Brand Equity as Dependent Variable)

**Variables**	**Step 1 (Controls) β**	**Step 2 (Leadership Predictors) β**	**Step 3 (Full Model) β**
Firm Size	.08	.07	.06
Firm Age	–.04	–.03	–.03
Ownership Structure	.05	.04	.03
Industry Controls	Included	Included	Included
Leader Gender	—	.18*	.21**
Gender Diversity Index	—	.24**	.27**
Financial Strategy	—	—	–.05 (ns)
Marketing Investment	—	—	.07**
Communication Style	—	—	.06*
**R^2**	.07	.16	.24
**ΔR^2**	—	.09	.08

Appendix E. Alternative Brand Equity Measures (Robustness Models)

**Predictor**	**Alt. Brand Measure A β**	**Alt. Brand Measure B β**	**Alt. Brand Measure C β**
Leader Gender	.19**	.17**	.18**
Gender Diversity Index	.25**	.23**	.24**
Financial Strategy	–.04 (ns)	–.03 (ns)	–.05 (ns)
Marketing Investment	.08**	.06*	.07**
Communication Style	.07*	.05*	.06*

Appendix F. Gender Distribution Across Executive Roles (N = 32)

Executive Role	Female	Male	Total
CEO	5	7	12
CFO	4	6	10
CMO	5	5	10
**Total**	**14**	**18**	**32**

Appendix G. Questionnaire

**Variable**	**Hypothesis Alignment**	**Item Code**	**Measurement Statement (5-point Likert scale)**	**Source**
**Financial Crisis Response Orientation (Austerity)**	H1a, H2a	FA1	During the crisis, top management emphasized aggressive cost-cutting measures.	Boyne & Meier (2009)
		FA2	Preserving short-term liquidity was prioritized over protecting long-term brand value during the crisis.	Boyne & Meier (2009)
		FA3	Marketing and brand-related expenditures were substantially reduced to manage financial risk during the crisis.	Rego et al. (2022)
**Marketing and Brand Investment Orientation**	H1b, H2b	MI1	Our organization continued investing in marketing activities despite financial constraints during the crisis.	Keller & Lehmann (2006)
		MI2	Marketing spending during the crisis was viewed as a strategic investment rather than a discretionary cost.	Hanssens et al. (2014)

		MI3	Brand-building and customer engagement initiatives were maintained or selectively increased during the crisis.	Rego et al. (2022)
**Crisis Communication Style**	H1c, H3	CC1	Senior leaders communicated openly and transparently with stakeholders during the crisis.	Coombs (2007)
		CC2	Crisis-related communications emphasized empathy and concern for customers and employees.	Eagly & Heilman (2016)
		CC3	Leadership communication during the crisis helped reassure stakeholders and maintain trust in the organization.	Zenger et al. (2009)
**Brand Equity Resilience**	H2a, H2b, H3, H4	BER1	Customer trust in our brand remained strong throughout the crisis.	Keller (1993)
		BER2	Our organization was able to retain customer loyalty despite the crisis.	Keller & Lehmann (2006)
		BER3	The perceived reputation and quality of our brand were resilient during the crisis period.	Rego et al. (2022)
